# Requirements and comparative analysis of reverse genetics for bluetongue virus (BTV) and African horse sickness virus (AHSV)

**DOI:** 10.1186/s12985-016-0574-7

**Published:** 2016-07-02

**Authors:** Piet A. van Rijn, Sandra G. P. van de Water, Femke Feenstra, René G. P. van Gennip

**Affiliations:** Department of Virology, Central Veterinary Institute of Wageningen UR (CVI), P.O. Box 65, Lelystad, 8200 AB The Netherlands; Department of Biochemistry, Centre for Human Metabolomics, North-West University, Potchefstroom, South Africa; Department of Infectious Diseases and Immunology, Faculty of Veterinary Medicine, Utrecht University, Utrecht, The Netherlands

**Keywords:** Reverse genetics, Orbivirus, *Reoviridae*, Bluetongue virus, African horse sickness virus, dsRNA, Genetic modification, Reassortment

## Abstract

**Background:**

Bluetongue virus (BTV) and African horse sickness virus (AHSV) are distinct arthropod borne virus species in the genus *Orbivirus* (*Reoviridae* family), causing the notifiable diseases Bluetongue and African horse sickness of ruminants and equids, respectively. Reverse genetics systems for these orbiviruses with their ten-segmented genome of double stranded RNA have been developed. Initially, two subsequent transfections of in vitro synthesized capped run-off RNA transcripts resulted in the recovery of BTV. Reverse genetics has been improved by transfection of expression plasmids followed by transfection of ten RNA transcripts. Recovery of AHSV was further improved by use of expression plasmids containing optimized open reading frames.

**Results:**

Plasmids containing full length cDNA of the 10 genome segments for T7 promoter-driven production of full length run-off RNA transcripts and expression plasmids with optimized open reading frames (ORFs) were used. BTV and AHSV were rescued using reverse genetics. The requirement of each expression plasmid and capping of RNA transcripts for reverse genetics were studied and compared for BTV and AHSV.

BTV was recovered by transfection of VP1 and NS2 expression plasmids followed by transfection of a set of ten capped RNAs. VP3 expression plasmid was also required if uncapped RNAs were transfected. Recovery of AHSV required transfection of VP1, VP3 and NS2 expression plasmids followed by transfection of capped RNA transcripts. Plasmid-driven expression of VP4, 6 and 7 was also needed when uncapped RNA transcripts were used. Irrespective of capping of RNA transcripts, NS1 expression plasmid was not needed for recovery, although NS1 protein is essential for virus propagation. Improvement of reverse genetics for AHSV was clearly demonstrated by rescue of several mutants and reassortants that were not rescued with previous methods.

**Conclusions:**

A limited number of expression plasmids is required for rescue of BTV or AHSV using reverse genetics, making the system much more versatile and generally applicable. Optimization of reverse genetics enlarge the possibilities to rescue virus mutants and reassortants, and will greatly benefit the control of these important diseases of livestock and companion animals.

## Background

Bluetongue (BT), African horse sickness (AHS), and epizootic haemorrhagic disease (EHD) are OIE listed arthropod borne animal diseases caused by the viruses in the genus *Orbivirus*, family *Reoviridae*. These viruses are spread by specific species of *Culicoides* biting midges. Outbreaks and geographic expansion of affected areas are associated to various factors, including climate change [1, 2], and the presence of competent biting *Culicoides* midges [3]. *Culicoides* species in countries with a moderate climate are competent insect vectors for BTV [4–6]. This implies that countries historically free of disease are probably at risk for BT and other midge borne diseases [7].

The prototype bluetongue virus (BTV) and African horse sickness virus (AHSV) are the best studied orbiviruses, reviewed in [8]. Orbiviruses are non-enveloped virus particles consisting of the subcore protein VP3, surrounded by serogroup specific protein VP7 (core), and the outer shell of the particle is formed by serotype specific protein VP2 and VP5. Orbiviruses contain a ten-segmented double stranded (ds) RNA genome (Seg-1–10). Replication proteins VP1, VP4 and VP6 are present in the virus particle [8]. In addition to structural proteins VP1-7, at least four non-structural proteins NS1 - 4 are expressed after infection of mammalian and insect cells but these are not present in the infectious virus particle.

After cell entry, the orbivirus particle is uncoated by removing VP2 and VP5 resulting in a transcription-active core particle that synthesizes capped messenger RNAs (mRNAs) of each genome segment. In addition to translation, these mRNAs act as template for dsRNA synthesis after recruitment from the cytoplasm into nascent virus particles. Together with replication proteins VP1, 4 and 6, and VP3, replication complexes are formed in virus inclusion bodies, and synthesize dsRNA in subcore particles which become core particles by addition of VP7 and virions by assembly of outer shell proteins VP2 and VP5. NS1 protein enhances protein expression from viral mRNAs [9]. NS2 recruits mRNA from the cytoplasm and is involved in the formation of VIBs [10]. NS3/NS3a is cytotoxic in mammalian cells but more prominent in insect cells, and is involved in non-lytic virus release from in particular insect cells [11, 12]. BTV NS3/NS3a also suppresses interferon induction in mammalian cells [13]. NS4 proteins of orbiviruses also seems to interfere with the host immune response and are important for virulence [14–18]. Recently, an additional protein expressed by Seg-10 has been discovered the functions of which are still unclear [19].

For a long time, research on orbiviruses has been hampered by the lack of reverse genetics systems. Functional studies on orbivirus proteins in infected cells were limited to natural variability in proteins and reassortants. First, one or two genome segments in BTV were replaced by incorporation of in vitro run-off RNA transcripts [20, 21]. Now, reverse genetics (RG) has been developed for different genera of the *Reoviridae* family, including for BTV and AHSV [22–25]. Recovery of reassortants showed that the constellation of the BTV genome is flexible [23, 26–31]. RG has also been used to study viral proteins [11, 14], as well as the role of RNA sequences in virus replication [32–35].

RG has also been used to develop very promising novel vaccines for orbiviruses. Disabled Infectious Single Cycle/Cell (DISC) vaccine or *in-trans* complemented BTV lacks the gene encoding essential VP6, but can be rescued and produced in constitutively VP6 expressing cells [36]. Disabled Infectious Single Animal (DISA) vaccine lacks the gene encoding non-essential NS3/NS3a which includes the DIVA principle [37], and has been produced in well-known cell lines similar to these for live-attenuated BT vaccines [11, 38]. Both novel BT vaccines have been explored for different serotypes by exchange of serotype determining genome segments using RG [39, 40]. Similarly, promising AHS DISA vaccine candidates has been developed using RG [12].

RG based on exclusively plasmids for in vivo run-off RNA transcripts has been developed recently for BTV [41]. Further, RG systems based on exclusively in vitro synthesized capped run-off RNA transcripts were modified by use of protein expression from plasmids for initial protein synthesis [24, 42]. Obviously, reverse genetics for orbiviruses is now used in functional studies as well as for vaccine development and the methods have changed over time. Here, we studied and compared the requirements to recover BTV and AHSV using reverse genetics in more detail.

## Methods

### Cell lines and viruses

BSR cells (a clone of baby hamster kidney cells [43]) were cultured in Dulbecco’s modified Eagle’s medium (DMEM; Invitrogen) containing 5 % foetal bovine serum (FBS), 100 IU/ml penicillin, 100 μg/ml streptomycin and 2.5 ug/ml Amphotericin B. Live-attenuated AHSV4LP has been generated approximately 50 years ago by different passages and plaque purification. The official passage number is HS32/62—10S-10BHK-3LP-7Vero [44]. BTV6/net08 is closely related to live-attenuated BT vaccine for serotype 6 [45, 46]. Reverse genetics of BTV6/net08 and AHSV4LP has been published [12, 26]. Virus stocks were obtained by infection of fresh BSR cells with a multiplicity of infection (MOI) of 0.1, and stored at 4 °C or -80 °C. Virus titres were determined by endpoint dilution and expressed as ^10^log TCID_50_/ml.

### Full length cDNA of genome segments

Full genome segment sequences of BTV6/net08 and AHSV4LP have been described (accession numbers: QG506472-QG506481 and accession numbers: KM820849-KM820858, respectively) [12, 26]. cDNAs of Seg-10[NS3/NS3a] of AHSV2 and AHSV3 have been described (accession numbers: KF860005 and KM886363). cDNAs of Seg-2[VP2] and Seg-6[VP5] of other AHSV serotypes have been described [12, 27, 40].

**Fig. 1:**
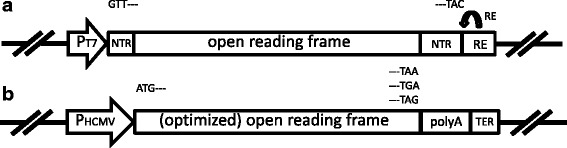
Schematic presentation of plasmids for run-off RNA transcription and protein expression. **a** cDNA of full genome segments were cloned between the promoter for DNA dependent RNA polymerase of bacteriophage T7 (P_T7_) and a recognition site of restriction enzyme (RE) *Sap*I, *Bsm*BI, or *Bsa*I to allow run-off RNA transcription. The appropriate RE was selected based on the absence of the recognition sites in the respective genome segment. Alternatively, internal sites were eliminated by silent mutations with respect to the translated amino acid sequence. **b** Authentic or open reading frames (ORFs) optimized for plasmid stability and eukaryotic expression (see materials and methods) were cloned between the immediate early promoter of human cytomegalovirus (P_HCMV_) and the polyA signal (polyA) and transcription terminator (TER)

Full genome segments were synthesized by Genscript corporation (Piscataway NJ, USA) and cDNAs were cloned in appropriate plasmids under control of the T7 promoter and 3’-terminally flanked by a restriction enzyme site suitable for full length run-off RNA transcription (Fig. 1a) [12, 23, 26]. Alternatively, internal sites of restriction enzymes were eliminated by synonymous mutations to allow full length run-off transcription, and expression of unchanged proteins. Mutated cDNAs of several genome segments were synthetically derived or constructed by standard procedures and have been described previously [12], except for Seg-10 ‘AUG total’ containing AUG- > GCC mutations for all 13 AUG codons of the NS3/NS3a ORF. delSeg-5 of AHSV contains an out-of-frame deletion of 79 nucleotides (nucleotide position 286-364). All plasmids were transformed and maintained in E. coli strain DH5α (Invitrogen) and were isolated using the High Pure Plasmid Isolation Kit (Roche) or the QIAfilter Plasmid Midi Kit (Qiagen). T7 RNA-polymerase driven run-off transcription was performed for capped RNAs with the mMESSAGE mMACHINE® T7 Ultra Kit, and for uncapped RNAs with the MEGAscript® T7 Kit as described [26]. Synthesized RNAs were purified using the MEGAclear kit (Ambion) according to manufacturer’s protocol and stored at -80 °C.

### Expression plasmids

Authentic open reading frames (ORF) of VP1, 3, 4, 6, 7, NS1 and 2 of AHSV4LP were inserted in expression plasmid HC pSMART or LC pSMART under control of the immediate early promoter of human cytomegalovirus by standard procedures (Fig. 1b) [47]. Plasmid with ORF-VP3 of AHSV4LP was unstable. A similar set of expression plasmids including for ORF-VP3 was constructed with the respective ORF optimized for eukaryotic expression and plasmid stability (Genscript corporation, Piscataway NJ, USA). Similarly, a set of expression plasmids with optimized ORF of VP1, 3, 4, 6, NS1 and 2 of BTV6/net08 was constructed. Expression plasmids were transformed and maintained as described for plasmids with full segment cDNAs.

### Immunoperoxidase monolayer assay (IPMA)

Protein expression was determined by immunoperoxidase monolayer assay (IPMA) according to standard procedures [48]. Transfected or infected monolayers of BSR cells were fixed with methanol/aceton (1:1) or 4 % paraformaldehyde in PBS. Immunostaining was performed by incubation with orbivirus directed antibodies followed by conjugated rabbit α-mouse serum or conjugated rabbit α-GP serum (DAKO). Monoclonal antibody (MAb) against BTV-VP7 (ATCC-1875) was used to detect BTV transfected/infected cells. For AHSV, MAb 10AE12 directed against VP5 and several MAbs were used directed against AHSV-NS3 (generous gifts from Paloma Rueda, Ingenasa, Spain). Guinea pig (GP) sera raised against baculovirus expressed VP2 protein (α-VP2 GP serum) of different AHSV serotypes have been published [49].

### Virus recovery from in vitro synthesized RNA transcripts

Reverse genetics (RG) using exclusively in vitro synthesized RNAs has been described for BTV [23, 26]. Briefly, monolayers of 10^5^ BSR cells per 2 cm^2^ were transfected twice with 600 ng RNA containing equimolar amounts of capped run-off RNA transcripts of six segments followed by transfection with a set of all 10 RNA transcripts. After 4 h, transfection medium was replaced by culture medium, and after 2 days culture medium was harvested. Virus recovery was determined by infection of fresh BSR monolayers. Cytopathogenic effect (CPE) and the immunoperoxidase monolayer assay (IPMA) confirmed virus recovery and virus identity. If negative, duplicate transfected wells were passed, screened for CPE and immunostained as described above. Sequential passaging of cells was repeated was repeated with immunostaining serving as indicator of virus replication. If immunostaining was no longer visible, attempts were repeated at least twice in order to conclude that virus recovery was considered unsuccessful.

### Virus recovery using expression plasmids and RNA transcripts

Reverse genetics (RG) using expression plasmids and in vitro synthesized RNAs is very similar to the method using exclusively in vitro synthesized RNAs [12, 26]. Briefly, BSR monolayers in 2 cm^2^ wells were first transfected with equimolar amounts of expression plasmids followed by transfection of the set of 10 RNAs as described above. A total of 600 or 300 ng DNA plasmids was transfected using 1.5 or 0.75 μl Lipofectamine™ 2000 (1:2.5; 1 mg/ml, Invitrogen) in Opti-MEM® I Reduced Serum Medium according to manufacturer’s conditions. The transfection of 10 RNAs was carried out as described above. For AHSV, the RNA transfection was the same but transfection medium was replaced after 22 h, and transfected monolayers were passed once. Medium was harvested after 2 days to infect fresh monolayers in order to confirm virus recovery. If negative, duplicate wells with transfected cells were processed as described for recovery of BTV.

### Recovery of virus mutants and reassortants

Mutants and reassortants of BTV and AHSV were generated according to the described and indicated methods (Table 1). Mutations and replaced genome segments were confirmed by sequencing as previously described [11, 12]. Briefly, entire Seg-10 was amplified using primers located at the ultimate ends followed by sequencing. Seg-2 and Seg-6 were partially amplified with appropriate primers and amplicons were sequenced. Consensus sequences were assembled for complete Seg-10 using Lasergene SeqMan Pro Software (DNASTAR, version 11) to verify the identity of introduced mutations. The identity of Seg-2 and Seg-6 was confirmed by alignment of the partially derived sequences.

**Table 1:** Overview of rescued AHSV4LP variants demonstrating the improvements of the RG method

Name	Seg of AHSV4LP^1^	Segment exchange^2^	600 ng	300 ng	300 ng	Virus rescue	CPE^3^ VP5	IPMA^4^	
			a	b	c			VP2	NS3
AHSV4LP	1–10	-	+	+	+	+	+	4	+
Serotyped 3	1, 3–5, 7–10	(2, 6) 3	-	+	nd	+	+	3	nd
Serotyped 6	1, 3–5, 7–10	(2/6) 6	-	+	nd	+	+	6	nd
AHSV1LP	1, 3–10	(2) 1	nd	+	nd	+	+	1	nd
AHSV2LP	1, 3–10	(2) 2	-	+	nd	+	+	2	nd
AHSV3LP	1, 3–10	(2) 3	-	+	nd	+	+	3	nd
AHSV4LP	1–10	-	+	+	+	+	+	4	+
AHSV5LP	1, 3–10	(2) 5	-	-	+	+	+	5	nd
AHSV6LP	1, 3–10	(2) 6	-	+	nd	+	+	6	nd
AHSV7LP	1, 3–10	(2) 7	nd	+	nd	+	+	7	nd
AHSV8LP	1, 3–10	(2) 8	nd	+	nd	+	+	8	nd
AHSV9LP	1, 3–10	(2) 9	nd	+	nd	+	+	9	nd
AHSV4LP	1–10	-	+	+	+	+	+	4	+
Without Seg-10	1–9	-	-	-	-	-	-	nd	nd
Seg-10 of AHSV2	1–9	(10) 2	nd	nd	+	+	+	nd	+
Seg-10 of AHSV3	1–9	(10) 3	-	-	+	+	+	nd	+
mutAUG1	1–9	(10) AUG1	+	nd	nd	+	+	nd	+
mutAUG1 + 2	1–9	(10) AUG1 + 2	nd	+	nd	+	Small	nd	+
mutAUG1 + 2 & STOPS	1–9	(10) AUG1 + 2&STOPS	nd	+	nd	+	Small	nd	+
delLD	1–9	(10) delLD	-	+	nd	+	Small	nd	+
delTMR1	1–9	(10) delTMR1	nd	nd	+	+	-	nd	-
delTMR2	1–9	(10) delTMR2	nd	nd	+	+	-	nd	±
AUG total	1–9	(10) AUGtotal	nd	nd	+	+	-	nd	-
AHSV1LP + (mutAUG1 + 2 & STOPS)	1, 3–9	(2) 1, (10) AUG1 + 2&STOPS	nd	-	+	+	Small	1	+
AHSV8LP + (mutAUG1 + 2 & STOPS)	1, 3–9	(2) 8, (10) AUG1 + 2&STOPS	nd	+	+	+	Small	8	+
AHSV8LP + (AUG total)	1, 3–9	(2) 8, (10) AUGtotal	nd	nd	+	+	-	8	-

(1) In total, a mixture of 600 or 300 ng in equal molar amounts of seven expression plasmids were transfected followed by transfection of indicated RNAs of Seg-1 to 10. (2) Exchanged Seg-2[VP2] or Seg-10[NS3/NS3a] are indicated. AHSV1LP to AHSV9LP and all Seg-10 mutants have been described previously, except for Seg-10 ‘AUG total’ containing AUG- > GCC mutations for all 13 AUG codons of the NS3/NS3a ORF. Expression plasmids contained authentic ORFs, except for ORF-VP3 (a & b), or all seven expression plasmids with ORFs optimized for plasmid stability and eukaryotic expression (c). In total, 600 or 300 ng expression plasmids were used. Virus rescue was determined and confirmed by IPMA and indicated as successful (+), unsuccessful (-), or not done (nd). (3) After immunostaining with α-VP5 MAb, plaque morphology was microscopically scored as normal CPE (+), small CPE (small), or immunostained plaques without CPE (-). (4) IPMA results with the respective α-VP2 GP serum is indicated by the serotype number, and with α-NS3 MAbs as positive (+), weak (±), negative (-) or not done (nd)

Plaque morphology (CPE) was determined as previously described [11, 12]. Briefly, BSR monolayers were infected at a multiplicity of infection (MOI) of 0.1. After incubation of infected monolayers under overlay medium (K1000 complete with 1 % methylcellulose), cells were fixed with methanol/aceton (1:1) and immunostained. Cytopathogenic effect (CPE) of separate plaques were compared, and semi-quantitated as normal (+), small or no CPE (-). Expression of proteins was studied by IPMA with MAbs or α-VP2 directed GP sera as described [12].

## Results

### Reverse genetics of AHSV4LP

Initially, recovery of AHSV4LP by transfecting in vitro synthesized capped run-off RNA transcripts on two subsequent days was studied according to reverse genetics (RG) method for BTV [26]. Unfortunately, ‘synthetic’ AHSV4LP could not be recovered (not shown). Briefly, the level of protein expression was studied after transfection of AHSV-VP7 capped RNA transcript. VP7 expression was also investigated by IPMA after transfection of capped run-off RNA transcripts of Seg-1[VP1], Seg-3[VP3], Seg-4[VP4], Seg-5[NS1], Seg-8[NS2], Seg-9[VP6], and Seg-7[VP7]. Transient expression and replication-driven enhancement of VP7 expression were expected, but immunostaining was hardly observed (not shown), suggesting that expression from capped run-off AHSV-RNA transcripts was too weak to initiate replication of AHSV.

Expression plasmids for ORFs of VP1, 3, 4, 6, 7, and NS1 and 2 were constructed to increase and to extend protein expression. However, the plasmid with VP3-ORF was not stable, and the ORF was successfully optimized for plasmid stability and eukaryotic expression. Results with VP7 expression plasmid showed a stronger expression compared to that from capped RNA (not shown). Transfection of expression plasmids of VP1, 3, 4, 6, 7, and NS1 and 2 followed by transfection of 10 in vitro synthesized capped run-off RNA transcripts resulted in recovery of AHSV4LP (Table 2). Apparently, the high expression from plasmids contributed to recovery of AHSV4LP.

**Table 2:** Requirements of expression plasmids and run-off RNA transcripts for AHSV recovery

Expression plasmids with authentic ORFs							RNA	Virus
VP1	VP3	VP4	VP6	VP7	NS1	NS2	Transcripts	Recovery
-	-	-	-	-	-	-	Capped	-
+	+	+	+	+	+	+	Capped	+
+	+	+	+	-	+	+	Capped	-
+	+	+	+	+	-	+	Capped	+
Expression plasmids with optimized ORFs							RNA	Virus
VP1	VP3	VP4	VP6	VP7	NS1	NS2	Transcripts	Recovery
+	+	+	+	+	+	+	Capped	+
-	+	+	+	+	+	+	Capped	-
+	-	+	+	+	+	+	Capped	-
+	+	-	+	+	+	+	Capped	+
+	+	+	-	+	+	+	Capped	+
+	+	+	+	-	+	+	Capped	+
+	+	+	+	+	-	+	Capped	+
+	+	+	+	+	+	-	Capped	-
+	+	-	-	-	-	+	Capped	+
+	+	+	+	+	+	+	Uncapped	+
+	+	-	+	+	+	+	Uncapped	-
+	+	+	-	+	+	+	Uncapped	-
+	+	+	+	-	+	+	Uncapped	-
+	+	+	+	+	-	+	Uncapped	+
+	+	-	-	-	-	+	Uncapped	-
+	+	+	+	+	+	+	minSeg-5	-
+	+	+	+	+	+	+	delSeg-5	-

AHSV expression plasmids in the first transfection mix are indicated. In the upper panel, non-optimized ORFs in expression plasmids were used, except for ORF-VP3. In the lower panels, expression plasmids with optimized AHSV-ORFs were used. Then, monolayers were transfected with 10 full length in vitro synthesized capped or uncapped run-off RNA transcripts, except for minSeg-5 RNA (without) and delSeg-5 RNA (Seg-5 RNA with a small out-of-frame deletion). Virus recovery was microscopically determined by CPE and confirmed by IPMA, and indicated as positive (+) or negative (-)

### Requirements for recovery of AHSV4LP

AHSV4LP was recovered with six expression plasmids containing authentic ORFs and optimized ORF for VP3, whereas recovery without VP7 expression plasmid was not successful. In contrast, NS1 expression plasmid was not required (Table 2). Lowering the amount of plasmids from 600 to 300 ng did not abolish recovery, while the damage to the monolayer by transfection was reduced. This shows the benefits of using plasmids with high protein expression levels.

Virus recovery using expression plasmids with optimized ORFs was studied (Table 2). Apparently, optimized ORFs encode functional proteins as AHSV4LP was recovered. Now, the contribution of individual expression plasmids in virus recovery was investigated. Plasmid-driven expressions of VP4, VP6, VP7, and NS1 was not essential for virus recovery using capped RNAs in the second transfection (Table 2). Note that plasmid-driven VP7 expression was essential for virus recovery with non-optimized ORFs in expression plasmids. Expression plasmids for VP1, VP3, and NS2 are essential for virus recovery. Indeed, transfection of these three expression plasmids followed by transfection of 10 capped RNA transcripts resulted in virus recovery (Table 2). However, all expression plasmids including VP7 were required in combination with transfection of uncapped RNA transcripts, except for NS1 expression plasmid. Regarding NS1 protein, virus recovery was not successful without entire Seg-5 RNA or with mutated Seg-5 lacking functional NS1 expression (Table 2). We conclude that Seg-5 RNA and the expressed NS1 protein are essential for AHSV replication, but are not required to initiate virus recovery in transfected cells.

### Requirements for recovery of BTV6/net08

Previously, RG for BTV using exclusively in vitro synthesized capped run-off RNA transcripts, have resulted in reassortants, mutants and variants with spontaneously introduced changes during rescue [27, 40]; [11, 33]. On the other hand, several modifications appeared not viable [32, 35, 40]. Here, expression plasmids with optimized ORFs of VP1, 3, 4, 6, and NS1 and 2 of BTV6/net08 were successfully used (Table 3). However, the improved RG method with expression plasmids did not result in rescue of previous nonviable BTV mutants (not shown). Subsequent studies showed that plasmid-driven expression of VP3, 4, 6 and NS1 are not required. This was confirmed by transfection of VP1 and NS2 expression plasmids and subsequent transfection of 10 capped run-off RNA transcripts. In combination with uncapped RNA transcripts, however, VP3 expression plasmid was also required for BTV recovery (Table 3).

**Table 3:** Requirements of expression plasmids and run-off RNA transcripts for BTV recovery

Expression plasmids with optimized ORFs						RNA	Virus
VP1	VP3	VP4	VP6	NS1	NS2	Transcripts	Recovery
-	-	-	-	-	-	Capped	-
+	+	+	+	+	+	Capped	+
-	+	+	+	+	+	Capped	-
+	-	+	+	+	+	Capped	+
+	+	-	+	+	+	Capped	+
+	+	+	-	+	+	Capped	+
+	+	+	+	-	+	Capped	+
+	+	+	+	+	-	Capped	-
+	-	-	-	-	+	Capped	+
+	+	+	+	+	+	Uncapped	+
+	-	+	+	+	+	Uncapped	-
+	+	-	+	+	+	Uncapped	+
+	+	+	-	+	+	Uncapped	+
+	+	+	+	-	+	Uncapped	+
+	-	-	-	-	+	Uncapped	-
+	+	-	-	-	+	Uncapped	-

BTV expression plasmids with optimized ORFs in the first transfection mix are indicated. Then, monolayers were transfected with 10 full length in vitro synthesized capped or uncapped run-off RNA transcripts. Virus recovery was microscopically determined by CPE and confirmed by IPMA, and indicated as positive (+) or negative (-)

Note that the requirement of expression plasmids for BTV recovery in combination with uncapped RNA transcripts is similar to the requirement of expression plasmids for AHSV recovery in combination with capped RNAs, namely VP1, VP3 and NS2 expression plasmids.

### Improved reverse genetics for AHSV demonstrated by rescue of mutants and reassortants

AHSV4LP was not recovered with RG using exclusively RNAs, whereas many mutants and reassortants were generated with expression plasmids followed by RNA transfection (Table 1). Throughout this study, the initial RG method for AHSV was slightly modified by lowering the amount of plasmids from 600 to 300 ng, and by using optimized AHSV-ORFs in expression plasmids. Many more virus variants were rescued with modified RG methods, indicating that these modifications obviously improved the method. For example, exchange of both Seg-2 and Seg-6 of serotypes 3 and 6 in AHSV4LP was initially not successful, whereas after lowering the amount of expression plasmid these were rescued. Similarly, single Seg-2 exchange of serotype 2, 3, and 6, and incorporation of delLD in AHSV4LP were rescued with this improved method (Table 1). Single exchange of Seg-10 of AHSV3 and Seg-2 of serotype 5 were only rescued by use of expression plasmids with optimized ORFs (Table 1). Many more mutants, reassortants and combinations of these were rescued with this method, since the suboptimal methods were no longer used (nd, Table 1). In practical terms, these results demonstrated the clear improvements of the RG method to regenerate AHSV variants.

Taken together, a set of nine single Seg-2 reassortants AHSV1LP – AHSV9LP was rescued as promising AHS vaccine candidates [12]. Further, a set of three Seg-10 reassortants representing three phylogenetic lineages of Seg-10 was rescued [50, 51]. Finally, we showed that NS3/NS3a knockout mutations can be combined with exchange of genome segments, like for Seg-2 representing the serotype of AHSV.

## Discussion

Reverse genetics (RG) has been developed for viruses of many families including those with double stranded RNA (dsRNA) segmented genomes. The latter includes members of *Birnaviridae* [52, 53], and different genera within the *Reoviridae* family [25]. RG for BTV, the prototype member of the *Orbivirus* genus has also been developed. This RG method followed two subsequent transfections of in vitro synthesized positive single stranded capped run-off RNA transcripts [23]. Capped RNAs expressing VP1, 3, 4, 6, and NS1 and NS2 were used for primary protein expression followed by transfection of a set of ten uncapped RNA transcripts to recover BTV containing these genome segments [26]. However, a similar RG system for AHSV was not successful [12]. These results indicate that recovery of BTV is more efficient than for AHSV.

Primary protein expression by the first transfection of RNA transcripts can be replaced by plasmid-driven expression for BTV recovery [42]. A similar method was also successful for AHSV [12, 24]. Apparently, RG by plasmid-driven expression followed by RNA transfection is more efficient than by transfection of exclusively RNA transcripts. Further, increase of the primary protein expression changes the amount of plasmids critical for AHSV recovery. Use of optimized-ORF expression plasmids, excludes the need of VP7 by which the requirements for RG of AHSV becomes similar to that of BTV (Tables 2 and 3).

NS1 expression by the first transfection is not essential for recovery of BTV and AHSV (Tables 2 and 3) [42]. NS1 increases protein synthesis from BTV RNAs [9], which explains that NS1 expression is not required for virus recovery with plasmid-driven protein expression. Expectedly, Seg-5 and its expressed NS1 protein of AHSV are essential for AHSV replication, since AHSV without Seg-5 or with mutated Seg-5 abolishing NS1 expression were not rescued (Table 2). In further agreement to BTV, initial VP7 expression is not required for AHSV recovery (Tables 2 and 3). AHSV-VP7 is however essential for RG with authentic ORF expression plasmids (Table 2). Others have suggested that VP7 stabilizes the BTV replication complex [42]. Similarly, it can be suggested that AHSV-VP7 is beneficial for AHSV recovery by stabilizing the replication complex as observed with suboptimal conditions, like the lower AHSV protein expression from authentic ORFs compared to optimized ORFs. These results indicate that initial NS1 and VP7 expression are not essential, but beneficial for virus recovery.

The minimal requirements of plasmid-driven expression differs between BTV and AHSV (Tables 2 and 3). VP1 and NS2 expression plasmids are essential for recovery of BTV and AHSV, irrespective of capped RNAs in the second transfection. VP3 expression plasmid is also essential, although BTV was recovered without VP3 expression plasmid in combination with capped RNA transcripts. Apparently, expression of VP1, VP3 and NS2 is essential for recovery of both orbiviruses, although delayed and lower expression of VP3 is sufficient for recovery of BTV.

Similar to the enhancement in recovery by VP7, we suggest that VP3 enhances orbivirus recovery by increase of the initial replication activity by stabilization of premature replication complexes and the formation of subcore particles. Consequently, suboptimal expression of VP1 and NS2 requires expression of VP3 and VP7 to efficiently recovery virus. In full agreement with this*,* in vitro reconstitution of infectious BTV core particles showed that VP1, VP4 and VP6 form a transcription complex that does not need other proteins for its activity [54]. NS2 recruits mRNA from the cytoplasm to the replication complex and forms virus inclusion bodies in vivo [10], and is therefore not needed for in vitro reconstitution of core particles.

Small amounts of VP4, 6 and 7 expressed from capped RNAs after 18–22 h are sufficient to initiate recovery of AHSV. These capped RNA transcripts are not needed for BTV recovery (Tables 2 and 3). Others have shown that VP6 is essential for BTV replication and acts early in the replication cycle of BTV [55]. We suggest that very low expression levels of these proteins from either uncapped RNA or after in vivo capping of transfected uncapped RNA transcripts are sufficient to initiate BTV recovery in our system. We have noticed that not all attempts with these conditions were successful suggesting that the efficiency of virus recovery was much lower.

Summarizing, VP1 and NS2 expression plasmids are essential for an optimized RG system of BTV and AHSV. VP3 expression plasmid is also essential for BTV recovery if uncapped RNAs are used. In contrast, only NS1 expression plasmid could be omitted in combination with uncapped RNAs for AHSV recovery. Generally, we conclude that recovery of BTV is more efficient than of AHSV. In order to increase the success rate for rescue of mutants and reassortants, the most optimal system is the combination of all seven expression plasmids with optimized ORFs and capped RNA transcripts for the second transfection. BTV1 and BTV8 variants were generated using BTV6 based ORF-optimized expression plasmids [35], and virulent AHSV5 was similarly recovered with AHSV4LP based expression plasmids (published elsewhere). We propose that one set of expression plasmids of the respective orbivirus species in combination with a set of ten even uncapped run-off RNA transcripts will be sufficient to quickly recover new orbivirus variants. A reproducible and efficient RG system is a prerequisite to conclude the lethality of mutations or genome constellations. Still, the essentiality of viral proteins or lethality of mutations could be conclusively demonstrated by use of *in trans* complementation followed by virus passage on ordinary cells [55].

Requirements for RG of BTV and AHSV showed many similarities as well as differences, likely caused by the difference in efficiency of virus recovery, but fundamental differences between these orbivirus species cannot be excluded. This comparative analysis of reverse genetics for orbivirus prototypes BTV and AHSV will contribute to development of RG methods for other orbiviruses, such as the OIE listed, notifiable epizootic haemorrhagic disease virus, but also for other orbiviruses and reoviruses. This will increase our understanding of the complex replication cycle and assembly process of these viruses with a segmented double stranded RNA genome.

## Conclusions

For optimal function of RG systems for the rescue of BTV and AHSV, a limited number of expression plasmids is required. Optimization of reverse genetics enlarge the possibilities to rescue virus mutants and reassortants.

## Abbreviations

AHSV, African horse sickness virus; BTV, bluetongue virus; DISA, Disabled Infectious Single Animal; DISC, Disabled Infectious Single Cycle/Cell; DMEM, Dulbecco’s modified Eagle’s medium; EHDV, epizootic haemorrhagic disease virus; FBS, foetal bovine serum; MOI, multiplicity of infection; mRNA, messenger RNA; ORFs, open reading frames; RG, reverse genetics
